# Real‐world effectiveness and safety of acalabrutinib in chronic lymphocytic leukaemia: Multicentre experience

**DOI:** 10.1111/bjh.70638

**Published:** 2026-07-02

**Authors:** Andrea Duminuco, Dario Leotta, Alessandro Allegra, Daniele Caracciolo, Laura Caruso, Annalisa Chiarenza, Vittorio Del Fabro, Valentina Di Martina, Amalia Figuera, Paolo Fabio Fiumara, Massimo Gentile, Vanessa Innao, Salvatrice Mancuso, Enrica Antonia Martino, Antonio Mosca, Giovanna Motta, Francesca Pedalino, Marco Rossi, Fabio Stagno, Giorgio Tona, Francesco Di Raimondo, Giuseppe Alberto Palumbo

**Affiliations:** ^1^ Dipartimento di Scienze Mediche Chirurgiche e Tecnologie Avanzate ‘G.F. Ingrassia’ University of Catania Catania Italy; ^2^ Hematology Unit With BMT AOU Policlinico ‘G.Rodolico‐San Marco’ Catania Italy; ^3^ Hematology Section A.O.U. Policlinico ‘G. Martino’, University of Messina Messina Italy; ^4^ Hematology Unit, Department of Clinical and Experimental Medicine University Magna Graecia Catanzaro Italy; ^5^ Faculty of Medicine and Surgery ‘Kore’ University of Enna Enna Italy; ^6^ Hematology Unit Ospedale ‘San Vincenzo’ Taormina Italy; ^7^ Hematology Unit, Department of Onco‐Hematology Azienda Ospedaliera Annunziata Cosenza Italy; ^8^ Department of Pharmacy, Health and Nutritional Science University of Calabria Rende Italy; ^9^ Hematology Unit Azienda Ospedaliera di Rilievo Nazionale e di Alta Specializzazione Garibaldi Catania Italy; ^10^ Hematology Unit Policlinico Universitario ‘P. Giaccone’ Palermo Italy; ^11^ Hematology Unit Azienda Universitaria Ospedaliera Renato Dulbecco Catanzaro Italy; ^12^ Hematology Unit Ospedale ‘Sant'Elia’ Caltanissetta Italy

**Keywords:** acalabrutinib, cardiovascular comorbidities, chronic lymphocytic leukaemia, real‐world experience, time‐to‐discontinuation

## Abstract

Chronic lymphocytic leukaemia (CLL) primarily affects elderly patients with comorbidities, yet real‐world data on acalabrutinib, a second‐generation Bruton's Tyrosine Kinase (BTK) inhibitor, remain limited outside clinical trials, particularly in Italy. This retrospective multicentre study evaluated acalabrutinib monotherapy across 10 Italian haematology centres (2021–2025), including 155 adults with CLL/small lymphocytic lymphoma (SLL) (median age 74 years, 44.5% ≥75 years, 35.5% relapsed/refractory, 59.4% cardiovascular comorbidities, 24.5% del17p/TP53). Primary end‐point was time‐to‐discontinuation (TTD ≥28 days interruption/progression/death); secondary included response rates (based on International Workshop on Chronic Lymphocytic Leukemia criteria), haematological recovery, overall survival (OS) and safety. Median TTD was not reached (16 months follow‐up), overall response rate 84.5% (32.9% clinical complete remission, reinforced by bone marrow evaluation in four cases), OS median not reached (78.7% alive), reporting rapid haematological recovery occurred (at 6 months haemoglobin 11.1–12.6 g/dL, platelets 146–160 × 10^9^/L, lymphocytes 57.5–12.3 × 10^9^/L; *p* < 0.001), uniform across subgroups (no TTD differences by age/line/cardiovascular comorbidities/biology; *p* > 0.05), without reduction of neutrophils (*p* = 0.48). Discontinuation was 20% (primarily due to adverse events), with low cardiovascular toxicity (one grade 3 atrial fibrillation). This study confirms acalabrutinib's durable effectiveness and tolerability in unselected real‐world CLL, above in patients with prior cardiovascular comorbidities, confirming clinical trial results also in frail elderly/high‐risk patients, supporting its broad adoption in routine care.

## INTRODUCTION

Chronic lymphocytic leukaemia/small lymphocytic lymphoma (CLL/SLL) is the most common adult leukaemia, with an incidence of 4–6 cases per 100 000 per year.^1^ In Italy, approximately 5 cases per 100 000 are diagnosed annually, predominantly in elderly, comorbid patients.^2^ Although 5‐year overall survival (OS) has improved substantially, it declines to approximately 75% in patients aged ≥75 years.^3^

The therapeutic landscape has been transformed by targeted agents, notably B‐cell lymphoma 2 (BCL‐2) and Bruton's Tyrosine Kinase (BTK) inhibitors, which have largely supplanted chemoimmunotherapy.^4^, ^5^ Acalabrutinib is a second‐generation covalent BTK inhibitor rationally designed to reduce off‐target kinase inhibition relative to ibrutinib, preserving efficacy while improving tolerability,^6^ and is active both as monotherapy and in combination with anti‐CD20 antibodies.^7^, ^8^

The phase III trials ELEVATE‐TN, ASCEND and ELEVATE‐R/R established acalabrutinib's efficacy across treatment‐naïve (TN) and relapsed/refractory (R/R) settings. In ELEVATE‐TN, acalabrutinib ± obinutuzumab reduced the risk of progression or death by ~90% and ~81% versus chlorambucil‐obinutuzumab, with 4‐year progression‐free survival (PFS) rates of 87.0%, 77.9% and 25.1%.^9^, ^10^ In ASCEND, 42‐month PFS was 62% vs. 19% for idelalisib‐rituximab or bendamustine‐rituximab.^11^, ^12^ ELEVATE‐R/R demonstrated non‐inferior PFS versus ibrutinib in high‐risk previously treated patients, with a superior cardiovascular safety profile.^13^, ^14^

However, pivotal trials enrol selected populations that poorly represent the elderly, frail, polymedicated patients encountered in routine practice. Real‐world analyses of acalabrutinib remain limited and available data are constrained by short follow‐up, heterogeneous capture and small sample sizes. Country‐specific real‐world evidence is therefore needed to characterize treatment patterns, effectiveness, safety and discontinuation in unselected populations.

## MATERIALS AND METHODS

### Patient selection and study design

This is a retrospective, observational, multicentre real‐world study across 10 Italian haematology centres. All adults (≥18 years) who initiated acalabrutinib monotherapy for CLL/SLL between January 2021 and October 2025 and received treatment for at least 1 month were eligible. The data cut‐off was December 2025. Secondary use of existing clinical documentation was performed in accordance with local regulations. Patient data were anonymized prior to analysis and informed consent was waived in accordance with local institutional policy.

Baseline was defined as the date of acalabrutinib initiation. Demographic variables, Eastern Cooperative Oncology (ECOG) performance status, comorbidities, cardiovascular history, renal function, biological features, cytogenetics and prior therapies were extracted. Treatment indication and response were defined per iwCLL 2018 guidelines.^15^ Beyond standard definitions, complete remission (CR) was interpreted as a clinical response assessed in routine practice rather than a strictly protocol‐defined iwCLL, since bone marrow (BM) biopsy was not systematically performed. Haematological parameters were assessed at baseline and at 6, 12 and 24 months of follow‐up.

### End‐points and statistical analyses

The primary end‐point was time‐to‐discontinuation (TTD), defined as the interval from treatment initiation to permanent discontinuation for any cause (adverse events [AEs], disease progression, Richter transformation or death), superimposable on event‐free survival (EFS). Treatment interruption lasting ≥28 consecutive days was considered a discontinuation. TTD was compared across predefined subgroups: treatment line (TN vs. R/R), age (<75 vs. ≥75 years), cytogenetic/biological risk (del(17p)/TP53 present vs. absent), immunoglobulin heavy chain variable (IGHV) mutational status and cardiovascular comorbidities (present vs. absent). Secondary end‐points included OS, drug utilization patterns (dose reductions, interruptions, reasons for treatment change) and safety (AEs leading to treatment modification).

Continuous variables are reported as median (range), categorical variables as frequencies and percentages. Mann–Whitney *U*, Pearson chi‐square or Fisher's exact test and Kruskal–Wallis tests were applied as appropriate. Survival outcomes were estimated by the Kaplan–Meier method and compared with the Wilcoxon test. Analyses were performed using MedCalc v22.003 (MedCalc Software, Belgium).

## RESULTS

### Baseline characteristics

Over a 4‐year period, 155 patients (91 males and 64 females) were enrolled, with a median age of 74 [45–89] years, 69 (44.5%) were aged ≥75 years.

At the time of CLL/SLL diagnosis, patients had a median age of 68 [40–87] years. Median time from diagnosis to initiation of acalabrutinib was 4 [1–21] years, reflecting a substantial disease history prior to enrolment.

From a biological standpoint (confirmed at the start of acalabrutinib treatment), del(17p) was identified in 21 (15.3%) of 137 patients with available data, TP53 mutation in 31 (20.2%) of 153 and the presence of both abnormalities in 14 cases. Overall, 38 (24.5%) patients were at high biological risk for del(17p)/TP53 mutation, 103 (66.5%) at low risk and in 14 cases it was not assessed. Unmutated IGHV status was documented in 98 (65.7%) of 149 tested patients.

Regarding prior CLL/SLL management, 100 (64.5%) patients were TN at inclusion, whereas 55 (35.5%) had prior treatment. Among previously treated patients, 43 (27.7%) had received one prior line, 11 (7.1%) two lines and 1 (0.7%) three. The most frequently administered prior regimens included chemoimmunotherapy rituximab‐based (R‐CHOP, FCR, R‐bendamustine, rituximab alone), chlorambucil, BTKi and venetoclax combined with obinutuzumab. One patient was treated with high‐dose chemoimmunotherapy for Richter's transformation.

Acalabrutinib was prescribed in accordance with iwCLL treatment initiation criteria. The primary indications for starting treatment included disease progression with massive/symptomatic splenomegaly or lymphadenopathy (65 patients, 41.9%), constitutional B‐symptoms (15, 9.7%) and alteration in peripheral blood count, such as anaemia, thrombocytopenia and progressive lymphocytosis (75, 48.4%). Among patients who had previously received a fixed‐duration treatment, the median time from the end of the previous line and initiation of acalabrutinib was 47 [1–175] months. Four patients were already treated with a BTKi (ibrutinib), interrupted due to intolerance/AEs.

A total of 139 (89.7%) patients with at least 5 × 10^3^/mmc lymphocytes were staged using the Binet and RAI staging systems. According to Binet, 26 (16.8%) were stage A, 52 (33.5%) stage B and 61 (39.4%) stage C. Based on RAI, 26 (16.8%) of patients were assigned to stage 0, while 113 (72.3%) were ≥1. Considering biology, the CLL‐IPI score, even if developed at diagnosis and not validated during follow‐up, was calculated for 141 patients with available combined molecular data, classifying 11 (7.1%) patients as low‐, 32 (20.6%) intermediate‐, 67 (43.2%) high‐ and 31 (20%) very high risk.

At the time of acalabrutinib initiation, hypertension was the most prevalent cardiovascular comorbidity, affecting 78 patients (50.3%), followed by arrhythmias, specifically atrial fibrillation (AF), paroxysmal supraventricular tachycardia and atrio‐ventricular block, which were reported in 6 patients (3.9%). Hypertensive heart disease and prior myocardial infarction were also overall present in seven patients (4.5%). When stratified by age, cardiovascular comorbidities affected 44 of 86 (51.2%) of patients <75 years and 45 of 69 (65.2%) of those ≥75 years.

All patients initiated acalabrutinib at 100 mg twice daily.

Other clinical and laboratory data are reported in Table 1.

**TABLE 1:** Clinical data at acalabrutinib initiation.

	155 patients (%) [range]
Median age (years)	74 [45–89]
Sex, male	91 (58.7)
Prior treatment lines	
None	100 (64.5)
1	43 (27.7)
2	11 (7.2)
3	1 (0.6)
IGHV (*available for 149 patients*)	
Mutated	51 (34.3)
Unmutated	98 (65.7)
del(17p) (*available for 137 patients*)	
No	116 (84.7)
Yes	21 (15.3)
TP53 (*available for 153 patients*)	
No	87 (79.8)
Yes	31 (20.2)
Hepato/splenomegaly	
No	117 (75.5)
Yes	38 (24.5)
CV risk factors	
No	63 (40.6)
Yes	92 (59.4)
Blood cell values	
Hb (g/dL)	11.1 [6.5–16.4]
PLT (×10^9^/L)	146 [3–393]
WBC (×10^9^/L)	63.73 [2.8–743]
Lymphocytes (×10^9^/L)	57.46 [0.97–685]
Neutrophils (×10^9^/L)	4.4 [0.2–69]
Serology	
LDH (U/L)	237 [96–1780]
B2M (mg/L)	4 [1–13]
Immunoglobulins	
IgG (mg/dL)	722 [10–4950]
IgM (mg/dL)	46 [5–2250]
IgA (mg/dL)	71 [1–2120]

Abbreviations: B2M, β2‐microglobulin; CV, cardiovascular; Hb, haemoglobin; LDH, lactate dehydrogenase; PLT, platelets; WBC, white blood cell.

### Baseline characteristics

**FIGURE 1:**
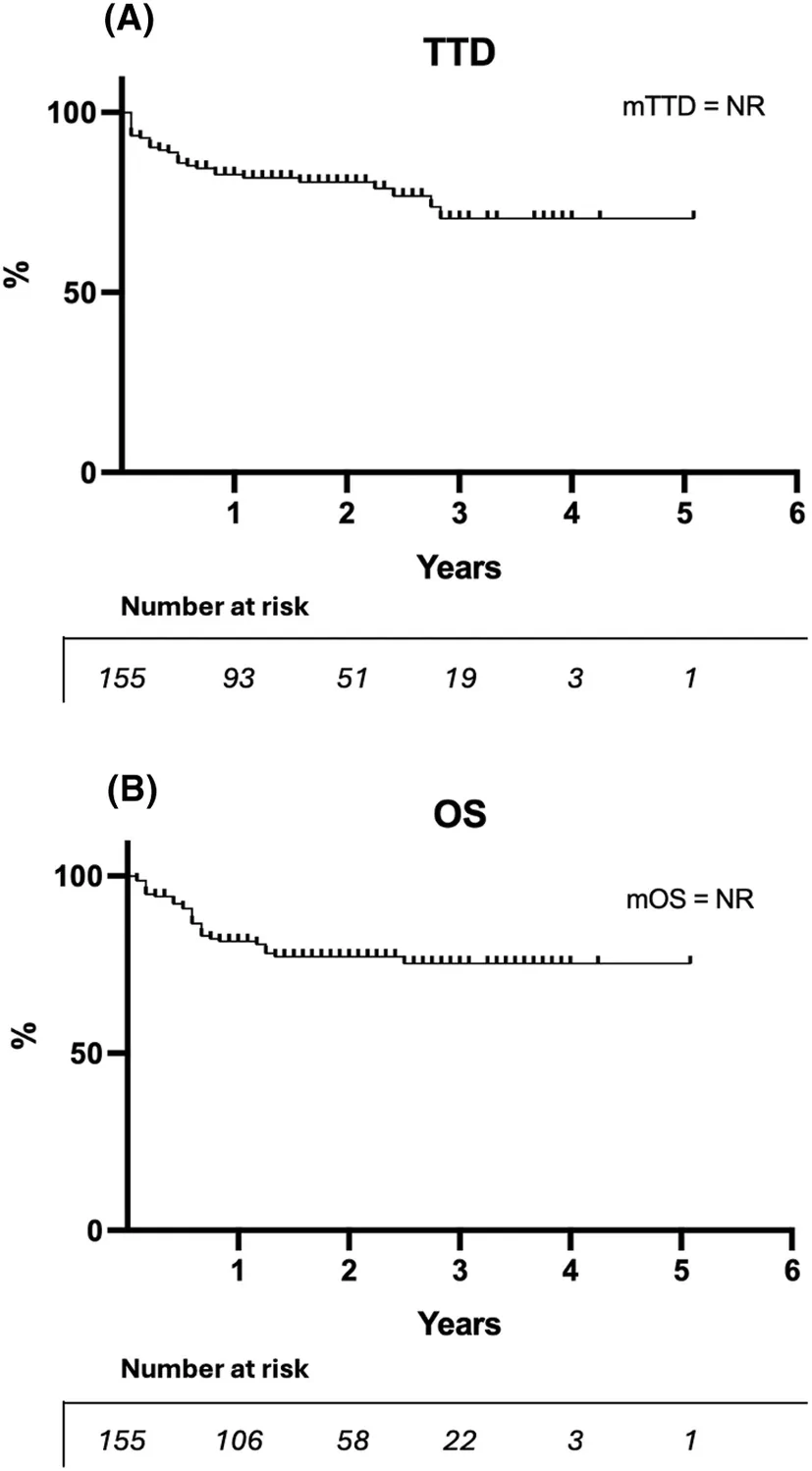
(A, B) Time‐to‐discontinuation (TTD—A) and overall survival (OS—B) for the entire cohort, as described in the text.

Median follow‐up was 16 [1–61] months, while median TTD was not reached (Figure 1). At the data cut‐off, 124 (80%) patients were receiving treatment and overall, 122 (78.7%) were alive. The median OS, calculated from treatment initiation to the most recent available data collection, was not reached (Figure 1B).

**FIGURE 2:**
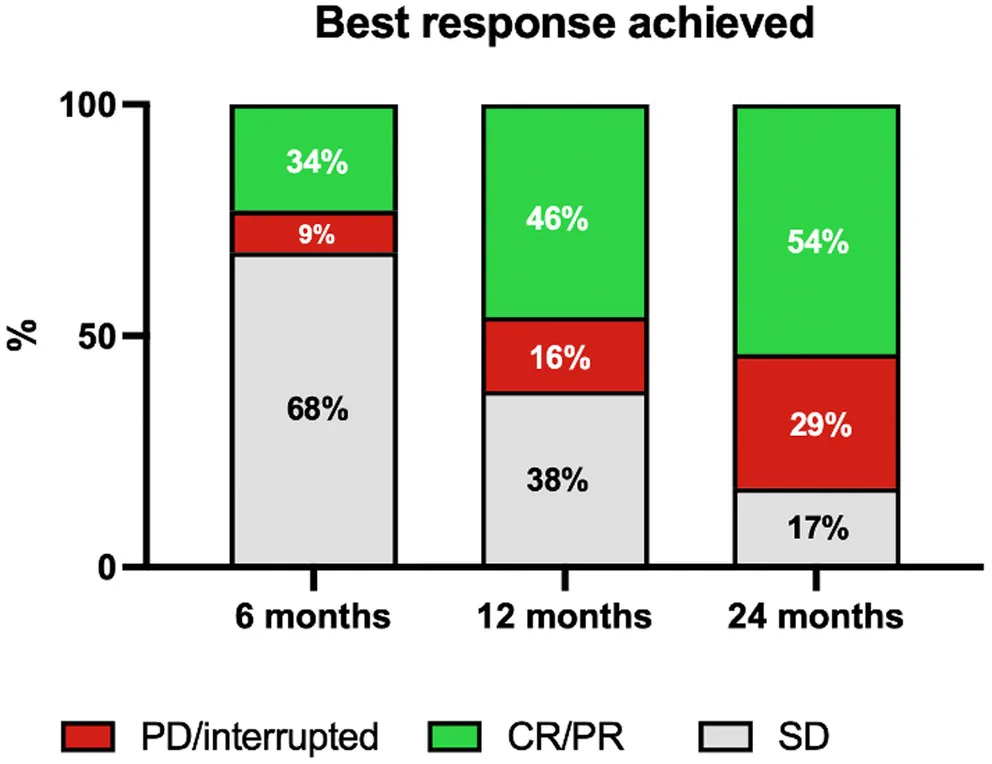
Rate of best response achieved, split in those who interrupted acalabrutinib due to progression of disease (PD) or adverse events (AEs) (in red), who reached complete/partial remission (CR/PR, in green) or those censored, in stable disease (SD, in grey) at one of the three different time points (overall 146 at 6 months, 123 at 12 and 45 at 12).

In the entire cohort, 51 patients (32.9%) achieved clinical CR. Among them, BM biopsy confirming remission was performed in four patients (2.5%). Secondarily, 80 (51.6%) achieved partial remission (PR). The remaining patients exhibited either stable disease (SD) (9, 5.8%) or progressive disease (PD) (5, 3.2%). The overall response rate (ORR) was 84.5%. The best response was reached and assessed after 12 [1–49] months of therapy, possibly due to the delay for repeat BM evaluation. The best response was not evaluable for 9 (5.8%) patients, mainly due to early discontinuation. Among patients who reached each time point (146, 123, 87, respectively), the CR/PR progressively improved from 34% at 6 months to 46% at 12 months and 54% at 24 months (Figure 2).

As reported in Table 2, the cohort was divided to compare those achieving a response (CR/PR) and those with SD/PD (plus those not evaluable for early discontinuation). A significant difference in age was observed, with 37.4% of patients in the CR/PR group being ≥75 years old, compared with 83.3% in the SD/PD group (*p* < 0.001), suggesting that older patients are more likely to have poorer treatment responses. Regarding RAI‐Binet staging, most patients in the CR/PR group were at advanced stages (75.6%), whereas a smaller proportion of patients in the SD/PD group were in this category (58.3%, not statistically significant). Moreover, there were no significant differences in the IGHV mutational status or the presence of del(17p)/TP53 between the two groups, indicating a similar distribution of biological alterations.

**TABLE 2:** Patients split according to best response achieved.

	CR/PR (*n* = 131)	SD/PD/NE (*n* = 24)	*p*
Age (years)			
≥75	49 (71)	20 (29)	<0.001
Sex			
Male	76 (83.5)	15 (16.5)	0.822
Stage RAI—BINET			
0—A	21 (80.7)	5 (19.3)	0.353
1–4—B–C	99 (87.6)	14 (12.4)	
IGHV			
Mutated	42 (82.4)	9 (17.6)	0.636
Unmutated	84 (85.7)	14 (14.3)	
del(17p)/TP53			
No	87 (84.5)	16 (15.5)	>0.999
Yes	32 (84.2)	6 (15.8)	
Anaemic/thrombocytopenic			
No	69 (88.5)	8 (11.5)	0.119
Yes	62 (79.4)	16 (20.6)	
Hepato/splenomegaly			
No	97 (82.9)	20 (17.1)	0.442
Yes	34 (89.4)	4 (10.6)	
Line of therapy			
Treatment naïve	82 (82)	18 (18)	0.353
Previously treated	49 (89.1)	6 (10.9)	
CV risk factors			
No	54 (85.7)	9 (14.3)	0.823
Yes	77 (83.7)	15 (16.35)	
Median time‐on‐acalabrutinib (months)	19 [1–61]	5 [1–14]	<0.001

Abbreviations: CR, complete remission (both clinical and histological); CV, cardiovascular; NE, not evaluable; PD, progression disease; PR, partial remission; SD, stable disease.

For baseline cytopenias (anaemia and thrombocytopenia), 47.3% of patients in the CR/PR group were affected, compared with 66.7% in the SD/PD group (*p* = 0.119). Hepatosplenomegaly did not differ significantly. It was found in 30% of the CR/PR group, compared with 16.7% in the SD/PD group (*p* = 0.442).

Lastly, the line of therapy did not have a significant impact, with 62.6% of CR/PR patients and 75% of the SD/PD group being TN. However, as expected, the median duration of acalabrutinib treatment differed markedly. The CR/PR group had a median time of 19 [1–61] months, whereas the SD/PD group had a much shorter median time of 5 [1–14] months.

**FIGURE 3:**
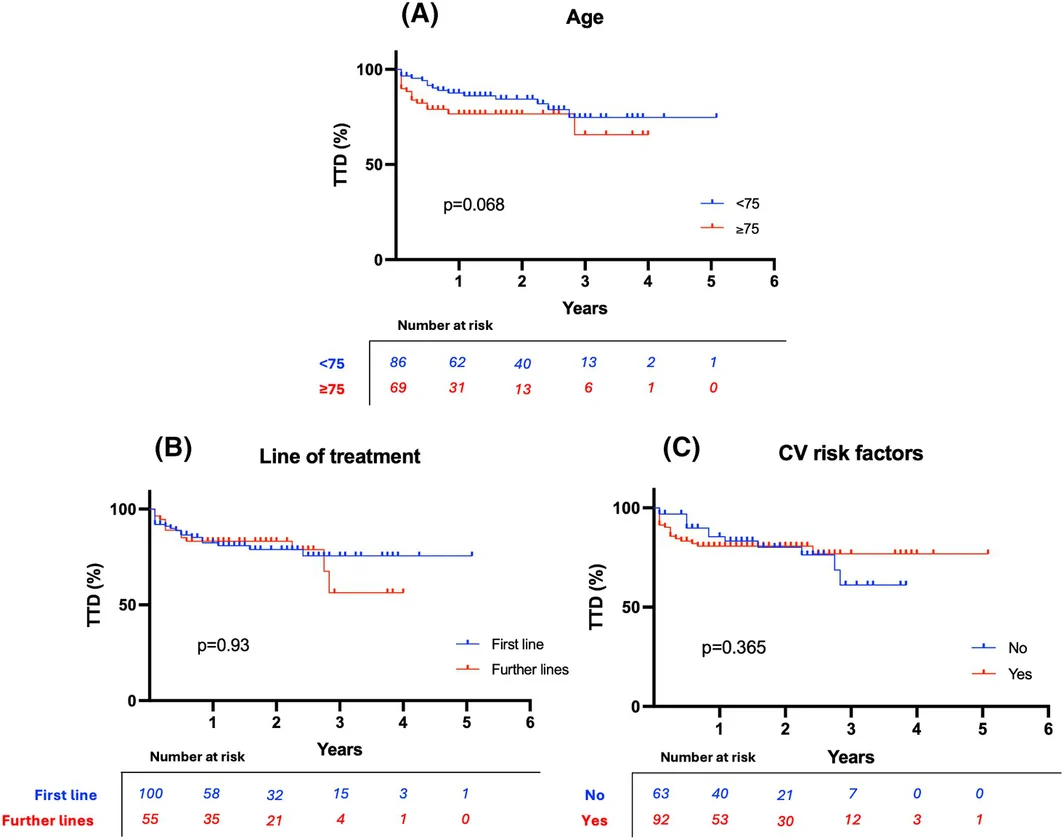
(A–C) Time‐to‐discontinuation (TTD), split according to age (A) line of treatment (B) and cardiovascular risk factor (C).

Kaplan–Meier survival analysis was conducted to assess TTD. Regarding age, a trend towards longer treatment duration was observed in patients <75 years, although this difference did not reach statistical significance (*p* = 0.068), indicating that age is associated with treatment outcomes, but the effect was not strong enough to establish a clear correlation in this cohort (Figure 3A).

Regarding treatment line, no significant difference was observed between first‐line and further (*p* = 0.93; Figure 3B).

First, when stratified by cardiovascular risk factors, there was no significant difference in treatment duration between patients with and without these comorbidities (*p* = 0.365), indicating that cardiovascular risk factors did not influence treatment duration in this cohort and that acalabrutinib can be used effectively (Figure 3C).

**FIGURE 4:**
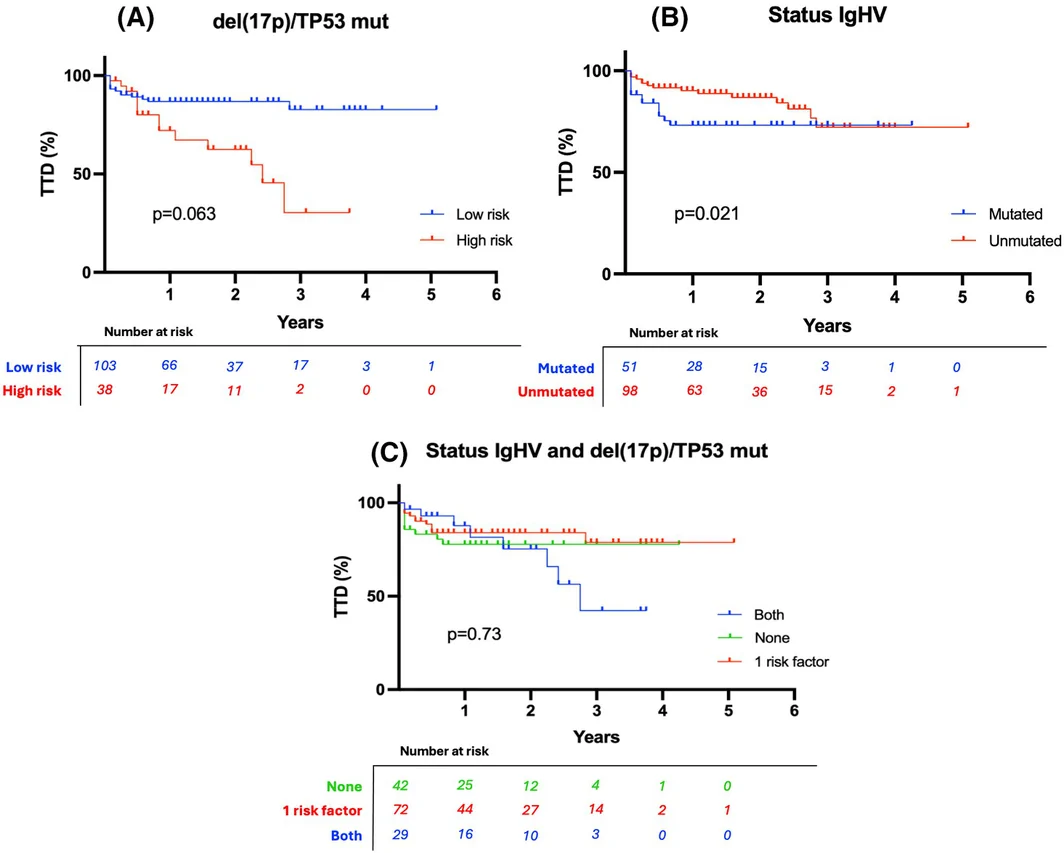
(A–C) Time‐to‐discontinuation (TTD), split according to del(17p)/TP53 mutation (A) status IGHV (B) and combined (C).

Patients with del(17p)/TP53 mutations tended to have a shorter TTD than those without these high‐risk genetic alterations, although this difference did not reach statistical significance (*p* = 0.063; Figure 4A).

On the other side, stratification by IGHV mutational status revealed a significant difference. Patients harbouring unmutated IGHV initially showed a higher EFS, but thereafter, the curve declined more rapidly compared to patients with mutated IGHV, whose survival remained more stable for a longer period, suggesting longer treatment durations (*p* = 0.021; Figure 4B).

Combining biological findings (IGHV and del(17p)/TP53 statuses), 3 groups (none, 1 or 2 risk factors) were identified for 143 patients with available data, with median TTD reached only for patients with both del(17p)/TP53 mutated and IGHV unmutated (33 months), but no statistically significant differences (*p* = 0.73; Figure 4C).

### Haematological parameters during follow‐up

At baseline, the median haemoglobin (Hb) level was 11.1 g/dL (range 6.5–16.4), showing a progressive change over follow‐up with median values of 12.6 g/dL (range 7.1–16.7) at 6 months, 13.1 g/dL (range 9.1–17) at 12 months and 13.6 g/dL (range 10.6–17.5) at 24 months. Platelet (PLT) counts followed a similar trend, with a baseline median of 146 × 10^9^/L (range 3–393) and median values of 160, 160 and 189 × 10^9^/L at 6, 12 and 24 months respectively.

White blood cell (WBC) counts demonstrated marked variation during treatment, with a baseline median of 63.73 × 10^9^/L (range 2.8–743) and subsequent medians of 15.4, 10.32 and 8.36 × 10^9^/L at 6, 12 and 24 months respectively. In parallel, the absolute lymphocyte count showed a progressive reduction over time, with a baseline median of 57.46 × 10^9^/L (range 0.97–685) decreasing to 12.3, 5.37 and 3.95 × 10^9^/L at the respective follow‐up time points.

Absolute neutrophil counts remained relatively stable throughout follow‐up, with median values of 4.4 × 10^9^/L (range 0.2–69) at baseline and 3.65, 3.8 and 3.95 × 10^9^/L at 6, 12 and 24 months.

Statistical comparisons between baseline and follow‐up time points demonstrated progressive recovery in Hb and PLT counts (*p* < 0.001). The same results were described for WBC and, specifically, lymphocytes, with a marked expected reduction (*p* < 0.001). Conversely, no significant change over time was reported for neutrophils (*p* = 0.48).

**FIGURE 5:**
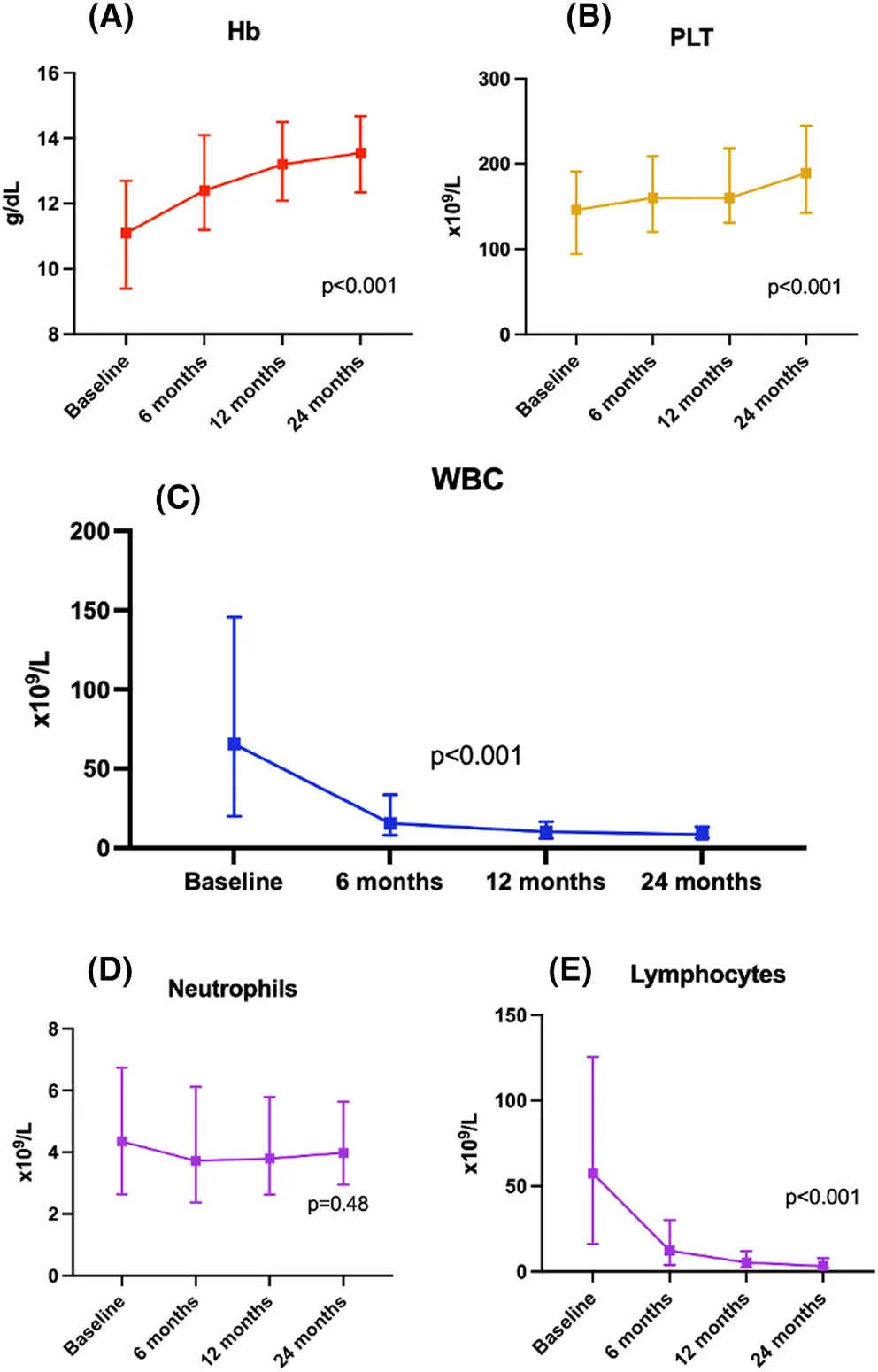
(A–E) Fluctuation in blood count values for haemoglobin (A) platelets (B) white blood cells (C) lymphocytes (D) and neutrophils (E) as described in the text.

A visual representation of full blood count fluctuation is reported in Figure 5.

### Safety, treatment interruptions and AEs

Overall, the treatment was well‐tolerated. 38.1% (59) of patients experienced at least one AE of any grade during the treatment period. The most common AEs were haematological (24, 15.8%), with 15 (9.7%) patients experiencing grade 3/4 neutropenia that required granulocyte colony‐stimulating factor. Anaemia and thrombocytopenia of grade 2/3 were reported in 2 (1.3%) patients, occurring after 1 and 8 months respectively.

Moving on non‐haematological AEs, no grade 4/5 AEs were reported. Among grade 3, five AEs were reported, specifically one bilateral pneumonia, three massive cutaneous haematomas and one case of AF. Other relevant grade 1/2 AEs were infections (seven, pneumonia and hepatitis C virus reactivation), musculoskeletal/cutaneous (seven, joint pain, skin rash), gastrointestinal (three, nausea, diarrhoea) and headaches (seven, all reported in the first days after acalabrutinib initiation). Focusing on common BTKi events, 5 cardiovascular AEs were reported, specifically 4 AF (twice in two cases) and 1 arterial hypertension, associated with 12 cases of spontaneous haematomas, exclusively cutaneous and gastrointestinal. Dose reductions (at any time points during follow‐up) were required in 29 (18.7%) patients, who continued treatment at a reduced dose of 100 mg once daily (from the standard 100 mg twice daily), primarily to improve tolerability and allowed most patients to remain on therapy without compromising overall treatment continuity.

As noted previously, treatment interruptions occurred in 31 (20%) patients, primarily due to AEs. The most common reasons for interruption included AEs of any type (15 cases) and patients' choice (4 cases). In three cases, discontinuations were due to non‐treatment‐related issues. Nine patients discontinued acalabrutinib due to loss of response, disease progression or resistance after a median of 13 [3–34] months.

Among those who interrupted acalabrutinib due to intolerance, loss of response or refractoriness, 15 received at least one further line of treatment, specifically 11 patients received rituximab and venetoclax, 2 patients received chemoimmunotherapy for Richter's transformation (followed by an allogeneic bone marrow transplant in 1 case and by epcoritamab in 1 other case) and 1 patient received chlorambucil as palliative care. Four patients restarted acalabrutinib after interruption achieving CR/PR at 16 [5–35] months from initiation.

## DISCUSSION

This multicentre real‐world study on acalabrutinib monotherapy in CLL/SLL contributes to the growing knowledge of real‐world evidence. While pivotal phase 3 trials, such as ELEVATE‐TN, ASCEND and ELEVATE‐RR, established acalabrutinib's efficacy and improved tolerability over ibrutinib in controlled, protocol‐driven environments, these studies systematically excluded older patients with significant comorbidities, cardiovascular risk factors or polypharmacy, who represent precisely the population predominant in routine clinical practice. Compared to ibrutinib, indirect comparisons suggest ibrutinib may outperform acalabrutinib in venetoclax combinations, though continuous monotherapy efficacy remains comparable.^16^

With a median age of 74 years and 44.5% aged ≥75 years, our population was notably older than in ELEVATE‐TN (median 70 years, 28% ≥75 years) or ASCEND (median 67 years), reflecting a worldwide ageing demographic.^9^, ^10^, ^11^, ^12^ The occurrence of cardiovascular comorbidities (affecting 59.4% at baseline), alongside non‐cardiovascular issues such as diabetes (14.3%) and prior solids (9%), usually excluded from clinical trials, reflected the use of acalabrutinib in clinical practice, where it is commonly used for more frail patients.

Focusing on real‐world data, the French NAOS study (nearly 500 patients; median age 75 years; 46%–59% ≥75 years) reported a similar elderly predominance and 55% cardiac comorbidities, but balanced TN and R/R distributions, whereas ours emphasized naïve cases.^17^

Australian real‐world data from a national reimbursement registry similarly demonstrated higher treatment adherence with acalabrutinib versus ibrutinib in R/R disease, with authors noting that unmeasured dose reductions may partly explain this signal,^18^ a pattern confirmed in our study, where dose modifications supported treatment continuity in frail patients. In this setting, the prospective CLL‐Frail specifically addressed acalabrutinib in patients aged ≥80 years and/or frail, demonstrating an ORR of 93.5% in the 46 patients receiving ≥3 cycles of treatment, meeting the primary end‐point of the trial (*p* < 0.001).^19^

These baseline features contextualize the study's observations of effectiveness. The absence of significant differences in TTD by treatment line, age or cardiovascular burden highlights consistent durability across vulnerabilities. This mirrors NAOS interim results (12‐month PFS 93.1% naïve, 87% R/R) and US real‐world datasets exploring BTKi outcomes and our median 16‐month follow‐up (80% ongoing) and rapid haematological recovery extend these benchmarks in a frailer cohort. Intriguingly, unmutated IGHV associated with paradoxically prolonged TTD (*p* = 0.021), while del(17p)/TP53 showed a non‐significant trend towards shorter duration (*p* = 0.063), aligning with pooled high‐risk data from ELEVATE‐RR and other acalabrutinib trials where efficacy held irrespective of adverse biology. The observed kinetics of IGHV status, with a faster initial TTD decline in unmutated IGHV CLL/SLL versus a delayed plateau in mutated cases, align with prior real‐world data on BTK inhibitors, in which IGHV‐unmutated disease (more B‐cell receptor‐dependent) exhibits more rapid cytoreduction.^20^ Multicentre analyses confirm prolonged BTKi‐associated lymphocytosis resolution in mutated IGHV, reflecting indolent biology with sustained long‐term benefit despite slower normalization, whereas higher apparent discontinuations in mutated IGHV may reflect indolent kinetics that mimic progression or cumulative AEs.^21^, ^22^ A preceding Italian series by Innocenti et al. in 98 high‐risk naïve patients reported no significant efficacy differences across biological subgroups, including del(17p)/TP53 and IGHV‐unmutated disease, consistent with our findings.^23^

The 20% overall discontinuation rate, driven primarily by AEs and progression, compares favourably to ibrutinib real‐world cohorts (30%–40% at comparable follow‐up) and to NAOS (22.7% at 12 months using an equivalent ≥28‐day interruption definition).^17^, ^24^, ^25^, ^26^

A large US propensity‐score matched analysis (TriNetX network) confirmed these signals: compared to acalabrutinib, ibrutinib was associated with higher all‐cause mortality (hazard ratio [HR] 2.269; 10.9% vs. 4.8%), higher rates of essential hypertension (HR 1.165) and haemorrhage (HR 1.304) and more Richter transformation events (HR 1.729), whereas headache and upper respiratory infections were more frequent with acalabrutinib.^27^

Haematological toxicities predominated in our cohort, while non‐haematological grade 3 events were sparse (one pneumonia, three massive cutaneous haematomas). Typical BTKi issues, such as early headaches (all mild), infections (7, mostly grade 1/2) and minor gastrointestinal disturbances, resolved without interrupting therapy. While discontinuation rates in our cohort (20% at 16 months) were low overall, they were comparable to those with ibrutinib. Recent multicentre studies further underscore second‐generation BTKi advantages, with zanubrutinib demonstrating lower 12‐month discontinuations (12.6% vs. 21.4% ibrutinib) and superior time to next treatment or death due to reduced AEs like AF, bleeding and infections, reinforcing acalabrutinib's position among selective BTKis for durable real‐world use.^28^

Several limitations inherent to this study's retrospective, chart‐based design should be acknowledged. First, as with all observational analyses, the non‐randomized treatment allocation introduces potential selection bias and confounding by indication, assuming that acalabrutinib may have been preferentially prescribed to patients perceived as suitable for its tolerability profile. The primary end‐point TTD, while widely adopted in real‐world BTKi analyses, does not constitute a validated surrogate for PFS or OS and should be interpreted as a composite measure of both efficacy and tolerability rather than a direct indicator of disease control, as discontinuation may be driven by AEs, patient choice or clinical judgement beyond disease progression, with lower‐grade AEs that may be underestimated. The missing data for biological and clinical variables (Fluorescence In Situ Hybridization [FISH]/del(17p) in 88.4%, TP53 in 98.7%, IGHV in 96.1%) and the relatively short median follow‐up of 16 months may reduce the ability to draw conclusions about long‐term outcomes and limit subgroup analyses to exploratory, hypothesis‐generating analyses. Finally, response assessment was based on routine clinical practice rather than protocol‐mandated central review, and BM biopsies were not systematically performed. As a consequence, CR rates likely overestimate strict iwCLL‐defined CR and should be interpreted with caution.

Nonetheless, the multicentre design, size and depth of clinical characterization distinguish this cohort from prior smaller analyses and support its generalizability to routine haematological practice.

## CONCLUSIONS

These data support expanded frontline and R/R use for elderly, comorbid or high‐risk patients who are underrepresented in trials. Prospective extensions and longer follow‐up will be essential to define long‐term OS, late cardiovascular toxicities and health‐economic trade‐offs.

## AUTHOR CONTRIBUTIONS

**Alessandro Allegra:** Investigation. **Valentina Di Martina:** Investigation. **Daniele Caracciolo:** Investigation. **Salvatrice Mancuso:** Investigation. **Vittorio Del Fabro:** Investigation. **Dario Leotta:** Data curation. **Annalisa Chiarenza:** Investigation. **Paolo Fabio Fiumara:** Investigation. **Amalia Figuera:** Investigation. **Laura Caruso:** Investigation. **Andrea Duminuco:** Conceptualization; investigation; methodology; formal analysis; writing – original draft; writing – review and editing; data curation. **Giorgio Tona:** Investigation. **Fabio Stagno:** Investigation. **Antonio Mosca:** Investigation. **Giovanna Motta:** Investigation. **Vanessa Innao:** Investigation. **Francesca Pedalino:** Data curation. **Marco Rossi:** Investigation. **Massimo Gentile:** Investigation. **Giuseppe Alberto Palumbo:** Conceptualization; writing – review and editing; supervision. **Enrica Antonia Martino:** Investigation. **Francesco Di Raimondo:** Conceptualization; supervision.

## FUNDING INFORMATION

The authors declare no funding sources.

## CONFLICT OF INTEREST STATEMENT

The authors declare no relevant conflicts of interest.

## Data Availability

The data that support the findings of this study are available from the corresponding author upon reasonable request.
